# Supplementary material for: Content-rich biological network constructed by mining PubMed abstracts
Source: BMC Bioinformatics. 2004 Oct 8;5:147. doi: 10.1186/1471-2105-5-147 (PMC528731; doi:10.1186/1471-2105-5-147)
Supplement: Additional File 2 — The original results of the above study (non-essential files are deleted to keep the file size under the limit set by BMC bioinformatics). [file 1471-2105-5-147-S2.bz2 › chilibotAdditionalFile2/dip10/216ID7744812E722/html/GRB2_DAG1.html]

 


 **GRB2** and **DAG1** 
  
Found 7 abstracts in PubMed, retrieved 7.  
 

 What does Google say? 
 PDF only 
| .edu only 

---

**Interactive relationship** (e.g. stimulation, inhibition, etc)

**Neutral relationship**- Analysis of the data obtained from SPR reveals a high affinity interaction K D approximately 240 nM between  **Grb2**  and the last 20 amino acids of the beta dystroglycan  [ **DAG1** ]  carboxyl terminus, which also contains a dystrophin binding site.  Ref: 10903901 Biochem Biophys Res Commun, 2000
- The  **Grb2**  beta dystroglycan  [ **DAG1** ]  interaction was inhibited by dystrophin in a range of concentration of 160 400 nM.  Ref: 10903901 Biochem Biophys Res Commun, 2000
- The specific dystroglycan  [ **DAG1** ]   **Grb2**  interaction may play an important role in extracellular matrix mediated signal transduction and or cytoskeleton organization in skeletal muscle that may be essential for muscle cell viability.  Ref: 7744812 J Biol Chem, 1995
- Characterization of the beta dystroglycan  [ **DAG1** ]  growth factor receptor 2  **Grb2**  interaction.  Ref: 10903901 Biochem Biophys Res Commun, 2000
- Affinity chromatography has also shown endogenous skeletal muscle  **Grb2**  interacts with beta dystroglycan  [ **DAG1** ] .  Ref: 7744812 J Biol Chem, 1995
- Immunoprecipitation experiments have demonstrated that  **Grb2**  associates with alpha beta dystroglycan  [ **DAG1** ]  in vivo in both skeletal muscle and brain.  Ref: 7744812 J Biol Chem, 1995
- This complex, in addition to beta dystroglycan  [ **DAG1** ] , was also found to contain  **Grb2**  and focal adhesion kinase p125FAK FAK.  Ref: 10098873 J Neurochem, 1999
- Protein binding assays with two  **Grb2**  mutants,  **Grb2**  P49L and  **Grb2**  G203R, which correspond to the loss of function mutants in the Caenorhabditis elegans sem 5, demonstrated that the dystroglycan  [ **DAG1** ]   **Grb2**  association is through beta dystroglycan  [ **DAG1** ]  C terminal proline rich domains and  **Grb2**  Src homology 3 domains.  Ref: 7744812 J Biol Chem, 1995
- beta Dystroglycan  [ **DAG1** ]  has now been expressed in vitro and shown to directly interact with  **Grb2** , an adapter protein involved in signal transduction and cytoskeletal organization.  Ref: 7744812 J Biol Chem, 1995
- **Grb2** , an adaptor protein involved in signal transduction and cytoskeleton organization, has been shown to bind beta dystroglycan  [ **DAG1** ] .  Ref: 10098873 J Neurochem, 1999
- SH3 domain mediated interaction of dystroglycan  [ **DAG1** ]  and  **Grb2** .  Ref: 7744812 J Biol Chem, 1995

**Non-interactive relationship** (e.g. studied together, co-existance, homology, etc.)

- We used surface plasmon resonance SPR, fluorescence analysis, and solid phase binding assay to measure the affinity constants between  **Grb2**  and the beta dystroglycan  [ **DAG1** ]  cytoplasmic tail.  Ref: 10903901 Biochem Biophys Res Commun, 2000
- Together, the data suggest that DA activated MAPK ERK via Ras, Raf 1 kinase, and DAG  [ **DAG1** ]  dependent PKC isoenzymes, but, importantly and contrary to the classical model, this pathway did not involve the  **Grb2**  Sos complex formation.  Ref: 11943676 Am J Physiol Lung Cell Mol Physiol, 2002
- We isolated both FAK and  **Grb2**  from synaptosomal extracts by chromatography on immobilized recombinant beta dystroglycan  [ **DAG1** ] .  Ref: 10098873 J Neurochem, 1999
- These results indicate that in addition to  **Grb2**  mediated activation of Ras, PLC gamma1 mediated DAG  [ **DAG1** ]  production is required for EGF and PDGF induced S phase entry and gene expression, possibly through activation of PKC.  Ref: 9418905 Mol Cell Biol, 1998
- At the synapses, the adaptor protein  **Grb2**  may mediate FAK beta dystroglycan  [ **DAG1** ]  interaction, and it may play a role in transferring information between the dystroglycan  [ **DAG1** ]  complex and other signaling pathways.  Ref: 10098873 J Neurochem, 1999
- Both  **Grb2**  SH3 domains bind beta dystroglycan  [ **DAG1** ]  but the N terminal SH3 domain binds with an affinity approximately fourfold higher than that of the C terminal SH3 domain.  Ref: 10903901 Biochem Biophys Res Commun, 2000
- Treatment of cells with diacylglycerol DAG  [ **DAG1** ]  or DAG  [ **DAG1** ]  and microinjected inositol, 5 triphosphate IP3, the products of activated PLC gamma1, did not stimulate cellular DNA synthesis by themselves but did suppress the inhibitory effects of the PLC gamma1 SH2 SH2 SH3 polypeptide but not the cell cycle block imposed by inhibition of the adapter protein  **Grb2**  or p21 Ras.  Ref: 9418905 Mol Cell Biol, 1998
- Blot overlay experiments demonstrate that alpha dystroglycan  [ **DAG1** ] , beta dystroglycan  [ **DAG1** ] , and syntrophins all bind  **Grb2** , the growth factor receptor bound adapter protein.  Ref: 11551227 Biochemistry, 2001
- The beta dystroglycan  [ **DAG1** ]   **Grb2**  interaction was investigated and a proline rich region within beta dystroglycan  [ **DAG1** ]  that binds  **Grb2**  src homology 3 domains identified.  Ref: 10903901 Biochem Biophys Res Commun, 2000
